# Predictive Factors of Resistance to High-Dose Steroids Therapy in Acute Attacks of Neuromyelitis Optica Spectrum Disorder

**DOI:** 10.3389/fneur.2020.585471

**Published:** 2020-11-12

**Authors:** Chuan Qin, Ran Tao, Shuo-Qi Zhang, Bo Chen, Man Chen, Hai-Han Yu, Yun-Hui Chu, Ke Shang, Long-Jun Wu, Bi-Tao Bu, Dai-Shi Tian

**Affiliations:** ^1^Department of Neurology, Tongji Medical College, Tongji Hospital, Huazhong University of Science and Technology, Wuhan, China; ^2^Department of Radiology, Tongji Medical College, Tongji Hospital, Huazhong University of Science and Technology, Wuhan, China; ^3^Department of Neurology, Mayo Clinic, Rochester, MN, United States

**Keywords:** NMOSD, attacks, high-dose steroids, response, predictor

## Abstract

High-dose steroids, the first-line therapy for acute attacks in neuromyelitis optica spectrum disorder (NMOSD), were ineffective in a proportion of NMOSD attacks. This study aimed to explore possible predictors of high-dose steroid resistance. Demographics and disease characteristics of acute attacks were compared between those who responded to high-dose intravenous methylprednisolone (IVMP) and those resistant to IVMP. In total, 197 attacks in 160 patients were identified in our NMOSD registry. Compared with responders, attacks resistant to high-dose steroids tended to have a higher proportion of previous history of immunosuppressive use (25.5 vs. 15.5%, *p* = 0.080). Significantly higher levels of proteins in the cerebrospinal fluid (CSF) were found in non-responders than in responders [485.5 (388–656) vs. 387 (291.5–532) mg/L, *p* = 0.006]. More active lesions were found in the brain stem of non-responders (8 attacks in 55, 14.5%), especially in the pons (7.3%) and medulla (14.5%), as opposed to responders (7 patients in 142, 4.9%). Multivariable logistic regression showed that resistance to high-dose steroid treatment was associated with previous immunosuppressant use [odds ratio (OR), 2.31; 95% confidence interval (CI) 1.002–5.34, *p* = 0.049], CSF protein level above 450 mg/L (OR 3.42, 95% CI 1.72–6.82, *p* < 0.001), and active lesions in the brainstem (OR 3.80, 95% CI 1.17–12.32, *p* = 0.026). In conclusion, NMOSD patients with previous use of immunosuppressants, higher levels of CSF protein, and active lesions in the brainstem are more likely to respond poorly to high-dose IVMP alone during an acute attack.

## Introduction

Neuromyelitis optica spectrum disorder (NMOSD) is a severe autoimmune disorder, characterized by recurrent attacks of optic neuritis and myelitis. A strong humoral response with autoantibodies against aquaporin-4 (AQP4) water channels on astrocytes has been identified as the main characteristic of NMOSD pathophysiology (1).

Adequate treatment of the attacks in NMOSD is crucial, considering the high probability of accumulating residual impairment from attacks, resulting in significant motor and visual disability in these patients (2–4). The mainstay therapy for acute attacks is high-dose intravenous methylprednisolone (IVMP), 1 g daily for three to seven consecutive days, followed by tapering off with oral prednisolone within 2 weeks (5–8). However, this combination targets the cellular part of the inflammatory process, which is only one aspect of NMOSD pathophysiology (9, 10). Some patients respond poorly to high-dose IVMP; therefore, aggressive treatments such as plasma exchange (PLEX) (9, 11), immunoadsorption (IA) (2), and intravenous immunoglobulin (IVIG) (12, 13) are routinely recommended as a rescue therapy or subsequent add-on therapy to target specific antibodies, complements, and several proinflammatory proteins (9). Therefore, it is crucial to identify the patients who are more likely to be resistant to high-dose IVMP therapy and initiate effective treatment as early as possible to reduce the risk of disability.

## Materials and Methods

### Patients and Study Design

**Figure 1:**
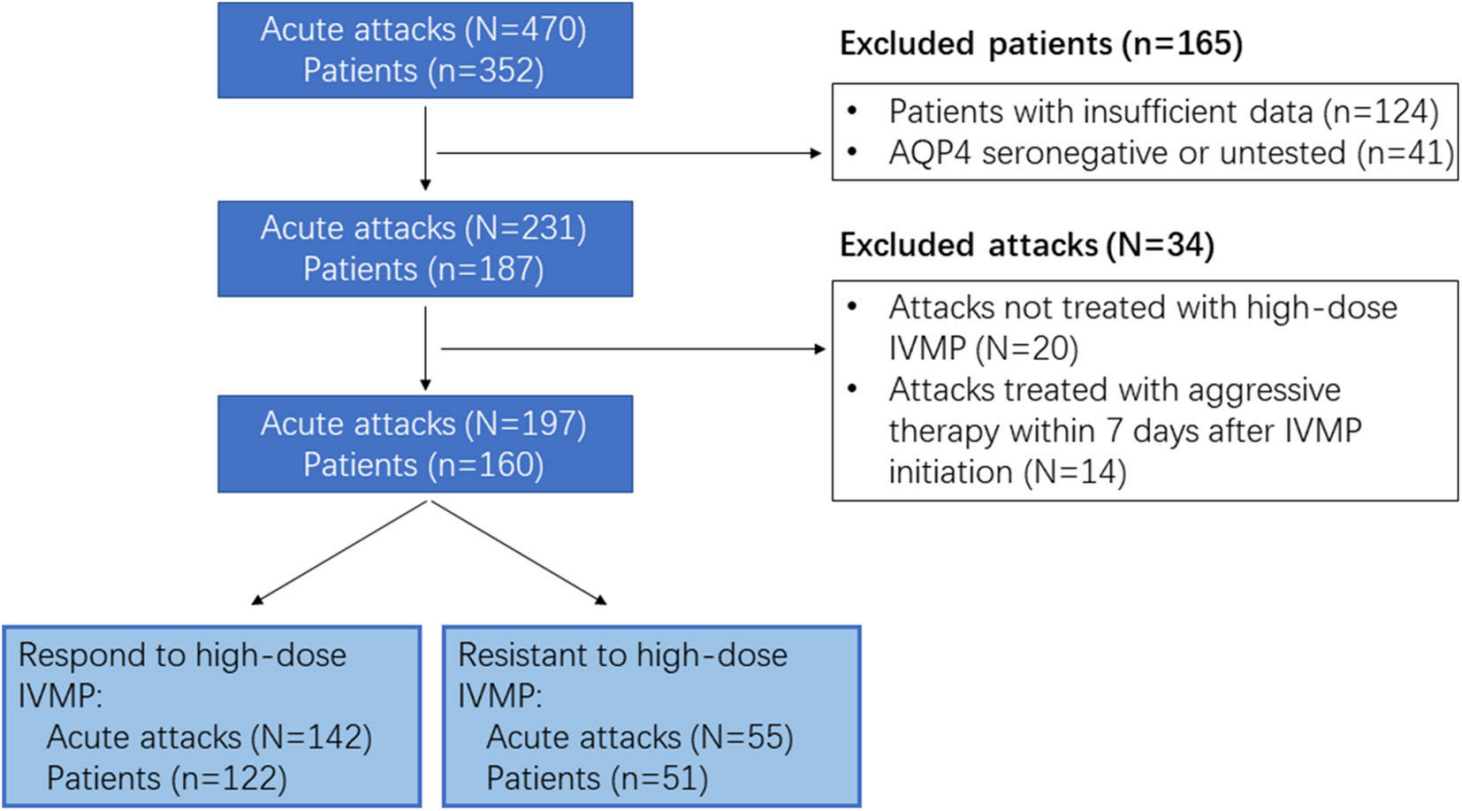
Flowchart of the present study. IVMP, intravenous methylprednisolone.

This study was based on a retrospective analysis of a single-center cohort of patients with NMOSD between August 2014 and June 2020 at the Tongji Hospital of Tongji Medical College, Huazhong University of Science and Technology, China. Data from all attacks in NMOSD patients, diagnosed per the 2015 criteria for NMOSD (14), were screened. AQP4-IgG were detected using a cell-based assay (CBA), as previously reported (15), using HEK293 cells expressing the human M23-AQP4 protein and an Alexa 488 conjugated secondary antibody (Thermo Fisher Scientific, Rockford, IL, USA), with 1:10, 1:32, 1:100, 1:320, 1:1000, or 1:3200 dilution. The farthest dilution yielding a positive result was recorded as the endpoint of positivity. AQP4-IgG seropositive patients who received pulse methylprednisolone treatment for an acute event of NMOSD were identified through the center's database. Patients with insufficient data were excluded from our study. A detailed study flowchart is shown in Figure 1. This study was approved by the Institutional Review Board of Tongji Hospital of Tongji Medical College, Huazhong University of Science and Technology (TJ-IRB20190502). Written informed consent was obtained from all patients or their authorizer.

### Definitions of Attack and Response

An attack was defined as an immune-mediated attack of the central nervous system (CNS), manifesting as new or worsening symptoms attributable to a new lesion seen on T2-weighted or contrast-enhanced MRI. All the acute attacks were treated with intravenous methylprednisolone at a dose of 1 g daily for five consecutive days, followed by halving every 3–5 days, and finally tapering off with small-dose oral prednisolone (20–30 mg/day).

The responses to IVMP were assessed on day 7 after the initiation of IVMP treatment (5). Patients were considered steroid responders if they showed improvement in strength or sensation of the affected limb, improvement in visual acuity, or improvement in bowel/bladder function.

A non-responder was defined as any of the following, as described before (1, 5, 16):

- for transverse myelitis (TM) attacks, having no improvement of ≥1 grade on the Medical Research Council (MRC) Scale for Muscle Strength of the affected limbs (17);
- for optic neuritis (ON) attacks, no improvement in visual acuity >2 lines in near chart vision, if the baseline visual acuity was ≥0.025, or one step better if the baseline visual acuity was worse than counting fingers (18);
- for area postrema syndrome (APS) attacks, the duration or frequency of the symptoms (nausea, vomiting, and hiccups) was unaltered or worsened, as evaluated by the clinicians (16);
- for attacks at other locations, no clinical improvement or worsening of the symptoms, as evaluated by the clinicians (1, 5).

### Data Collection

Patients were classified according to their response to IVMP. The data collected through chart review included demographics and disease characteristics for all patients. The Extended Disability Status Scale (EDSS) score was prospectively obtained to ensure a reliable analysis in our center. EDSS was also collected retrospectively from available records for the following time periods: at admission (pretreatment) and 7 days after the initiation of steroid treatment. Laboratory data, including anti-AQP4 status, cerebrospinal fluid (CSF) findings, and MRI findings (a new lesion on T2-weighted or contrast-enhanced MRI) were compared between the groups.

### Statistical Analysis

Data are presented as medians with interquartile range (IQR) values and percentages. Categorical variables were compared using the χ^2^ test. Continuous variables were compared using the Mann–Whitney *U*-test. Variables with two-tailed *p* < 0.05 were considered significant. Parameters approaching a significant difference (*p* < 0.1) in the comparison between respondent and non-respondent attacks were considered candidate parameters for predictive factors. Predictor exclusion was limited to parameters with more than 10% missing rate to minimize the bias of the regression coefficient. The missing values were imputed by the expectation-maximization method using SPSS statistical software, version 22 (IBM, Armonk, NY, USA). The candidate parameters included previous use of immunosuppressive agents, CSF cells, CSF proteins, and presentation of active lesions in the brain stem on MRI. Sex and age at the time of the attacks were treated as covariates and included in the final analysis. The potential risk factors associated with steroid resistance were analyzed using logistic regression. Each candidate variable was treated as a categorical variable and first fitted with univariate models one at a time to estimate the unadjusted OR and then included in the multivariable model.

## Results

### Patient Characteristics

A total of 197 attacks from 160 patients were included in the study. Clinical characteristics are described in Table 1. The majority of attacks were in women (171/197, 86.8%), with 122 (85.9%) responders and 49 (89.1%) non-responders. There were no significant differences in sex or age at the time of attacks between the two groups.

**Table 1:** Demographics and baseline characteristics of patients with NMOSD.

	**All attacks**	**Responders**	**Non-responders**	***p*-value**
**Baseline characteristics**				
Attacks no.	197	142	55	
Female, *n* (%)	171 (86.8)	122 (85.9)	49 (89.1)	0.370
Age at attacks, year, median (IQR)	45 (31–52)	44 (31–51)	47 (34–52)	0.172
**Manifestations of attacks**, ***n*** **(%)**				
ON	41 (20.8)	29 (20.4)	12 (21.8)	0.485
Unilateral	26 (13.2)	19 (13.4)	7 (12.7)	0.555
Bilateral	15 (7.6)	10 (7.0)	5 (9.1)	0.412
TM	95 (48.2)	70 (49.3)	25 (45.5)	0.373
Simultaneous ON and TM	28 (14.2)	19 (13.4)	9 (16.4)	0.370
ADEM or ADEM-like	25 (12.7)	17 (12.0)	8 (14.5)	0.393
Area postrema syndrome	8 (4.1)	7 (4.9)	1 (1.8)	0.293
Time from onset to IVMP initiation, days, median (IQR)	13 (7–31)	14 (7–29.75)	12 (7–32.5)	0.826
Previous use of immune suppressive drugs, *n* (%)	36 (18.3)	22 (15.5)	14 (25.5)	0.080
**Clinical status**				
Number of attacks pre-enrollment, median (IQR)	1 (0–2)	1 (0–2)	0 (0–2.5)	0.541
Baseline EDSS, median (IQR)	0 (0–2)	0 (0–2)	1 (0–3)	0.109
Pre-treatment EDSS, median (IQR)	4 (3–6)	4 (3–5.75)	5 (3–7)	0.118
7 days post-IVMP EDSS, median (IQR)	3 (2.75–5)	3 (2–4)	5 (4–7)	<0.001

*Data are presented as mean (SD), median (IQR), and n (%). p values were calculated from the comparison between responders and non-responders using the χ^2^ test, Fisher's exact test (for categorical variables), t-test, or Mann–Whitney U-test (for continuous variables). Pretreatment EDSS refers to EDSS at admission, before high-dose steroid therapy initiation. Responders refer to patients who responded to high-dose steroid therapy. Non-responders refer to patients who were resistant to high-dose steroid therapy. NMOSD, neuromyelitis optica spectrum disorder; SD, standard deviation; IQR, interquartile range; ON, optic neuritis; TM, transverse myelitis; ADEM, acute disseminated encephalomyelitis; EDSS, Extended Disability Status Scale; IVMP, intravenous methylprednisolone*.

In the 197 attacks, the most common manifestations at onset were transverse myelitis (TM) (48.2%) and optic neuritis (ON) (20.8%). In the 142 attacks that responded to steroids, the following presentations were recorded in the registry: 29 ON (19 unilateral and 10 bilateral), 70 TM, 19 simultaneous TM and ON, 17 acute disseminated encephalomyelitis (ADEM) or ADEM-like attacks, and 7 area postrema syndromes. In the 55 non-responding attacks, 12 presented with ON (7 unilateral and 5 bilateral), 25 TM, 9 simultaneous TM and ON, 8 ADEM or ADEM-like, and 1 area postrema syndrome. There were no significant differences in attack characteristics between the two groups (Table 1).

The median time interval from attack onset to IVMP initiation was 13 days [interquartile range (IQR) 7–13], with no difference between attacks responding to IVMP and non-responders. Compared with responders, attacks resistant to steroids tended to have a higher proportion of previous immunosuppressant use (25.5 vs. 15.5%, *p* = 0.080), although no difference in pre-enrollment attacks was found between the two groups. Clinical status (baseline EDSS and pretreatment EDSS) showed that severity and disability before attacks and at the time of attacks tended to be more severe in patients resistant to steroids [median 1 (IQR 0–3) vs. 0 (0–2), *p* = 0.109; 5 (3–7) vs. 4 (3–5.75), *p* = 0.118, respectively], compared with attacks responding to steroids, although the difference was not significant. EDSS at 7 days post-treatment for non-responders was significantly more severe than in responders [5 (4–7) vs. 3 (2–4), *p* < 0.001].

### AQP4-IgG Titer and CSF Findings

The laboratory findings, including the AQP4-IgG titer in serum and CSF testing, are shown in Table 2. No significant difference in AQP4-IgG titer was found between the IVMP responders and non-responders. The attacks resistant to steroids tended to have higher levels of cell count and albumin quotient in the CSF [7 (1–26) vs. 2 (0–14) 10^6^/L, *p* = 0.059; 6.9 (5.1–10.7) vs. 5.8 (4.1–8.5), *p* = 0.102, respectively], than the responders. Significantly higher levels of proteins in the CSF were found in non-responders than in responders [485.5 (388–656) vs. 387 (291.5–532) mg/L, *p* = 0.006]. Other findings, including intrathecal synthesis of IgG and oligoclonal bands in CSF, showed no significant differences between the two groups.

**Table 2:** Laboratory findings of patients with NMOSD.

	**Normal range**	**All attacks (*n* = 197)**	**Responders (*n* = 142)**	**Non-responders (*n* = 55)**	***p*-value**
AQP4 Ab titer	0	100 (32–320)	100 (32–320)	100 (26.5–320)	0.368
**CSF findings**					
Cell (10^6^/L)	0–8	3 (0–19)	2 (0–14)	7 (1–26)	0.059
CSF protein (mg/L)	150–450	412 (313.5–579)	387 (291.5–532)	485.5 (388–656)	0.006
Albumin quotient	<10	6.25 (4.2–8.6)	5.8 (4.1–8.5)	6.9 (5.1–10.7)	0.102
IgG index	0	0.7 (0.6–0.8)	0.7 (0.6–0.8)	0.7 (0.6–0.8)	0.346
Oligoclonal bands, *n* (%)	0	0 (0–0)	0 (0–0)	0 (0–0)	0.528

*Data are median (IQR), and n (%). p-values were calculated from the comparison between responders and non-responders using the χ^2^ test, Fisher's exact test (for categorical variables), t-test, or Mann–Whitney U-test (for continuous variables). Responders refer to patients who responded to high-dose steroid therapy. Non-responders refer to patients who were resistant to high-dose steroid therapy. NMOSD, neuromyelitis optica spectrum disorder; IQR, interquartile range; CSF, cerebrospinal fluid*.

### MRI Findings

New lesions on T2-weighted or contrast-enhanced MRI were counted and compared between the two groups. The percentage of patients with active lesions in different locations in the two groups is summarized in Table 3. More active lesions were found in the brain stem of non-responders (14.5%), especially in the pons (7.3%) and medulla (14.5%), as opposed to only 4.9% in the responders. There were no gadolinium-enhanced features in the optical nerve, spinal cord, or other brain regions, including the cerebrum and cerebellum that could reliably differentiate the two groups.

**Table 3:** Location of active lesion in patients with NMOSD.

	**No. (%)**			
	**All attacks (*n* = 197)**	**Responders (*n* = 142)**	**Non-responders (*n* = 55)**	***p*-value**
Optic nerve	23 (11.7)	18 (12.7)	5 (9.1)	0.333
**Brain**				
Cerebrum	6 (0.3)	4 (2.8)	2 (3.6)	0.536
Cortical gray matter/juxtacortical white matter	3 (1.5)	2 (1.4)	1 (1.8)	
Periventricular white matter	4 (2.0)	3 (2.1)	1 (1.8)	
Corpus callosum	1 (0.5)	1 (0.7)	0 (0.0)	
Brain stem	15 (7.6)	7 (4.9)	8 (14.5)	0.028
Midbrain	4 (2.0)	2 (1.4)	2 (3.6)	
Pons	6 (3.0)	2 (1.4)	4 (7.3)	
Medulla	13 (6.6)	5 (3.5)	8 (14.5)	
Cerebellum	3 (1.5)	2 (1.4)	1 (1.8)	0.628
**Spinal cord**					
Cervical	58 (29.4)	42 (29.6)	16 (29.1)	0.547
Thoracic	43 (21.8)	30 (21.1)	13 (23.6)	0.419
Length of active lesions on spinal cord, segments, median (IQR)	0 (0–3)	0 (0–3)	0 (0–4)	0.657

*Data are median (IQR), and n (%). p-values were calculated from the comparison between responders and non-responders using the χ^2^ test and Fisher's exact test. Responders refer to patients who responded to high-dose steroid therapy. Non-responders refer to patients who were resistant to high-dose steroid therapy. NMOSD, neuromyelitis optica spectrum disorder*.

### Predictive Factors of High-Dose Steroid Response

We performed an exploratory analysis of potentially independent predictors of high-dose steroid response (Table 4). In the univariate analysis, IVMP resistance was associated with age over 45 years old at the time of attacks (OR 2.05, 95% CI 1.09–3.86, *p* = 0.026), CSF protein level above 450 mg/L (OR 3.78, 95% CI 1.96–7.32, *p* < 0.001), and active lesions in the brainstem (OR 3.28, 95% CI 1.13–9.55, *p* = 0.029). In multivariable logistic regression, adjusted for age and sex, resistance to high-dose steroids was associated with previous use of immunosuppressive agents (OR 2.31, 95% CI 1.002–5.34, *p* = 0.049), CSF protein level above 450 mg/L (OR 3.42, 95% CI 1.72–6.82, *p* < 0.001), and active lesions in the brainstem (OR 3.80, 95% CI 1.17–12.32, *p* = 0.026).

**Table 4:** Logistic regression analysis of steroid resistance based on data at admission in our cohort.

	**Univariate analysis**		**Multivariable analysis**	
	**OR (95% CI)**	***p*-value**	**OR (95% CI)**	***p*-value**
Age at time of attacks, >45 vs. ≤ 45 years	2.05 (1.09–3.86)	0.026	1.59 (0.80–3.16)	0.185
Sex, female vs. male	1.34 (0.51–3.53)	0.556	1.76 (0.60–5.13)	0.301
Previous use of immune suppressive drugs, yes vs. no	1.86 (0.87–3.98)	0.108	2.31 (1.002–5.34)	0.049
CSF cell, >8 vs. ≤ 8 × 10^6^/L	1.43 (0.76–2.69)	0.274	0.99 (0.48–2.05)	0.977
CSF protein, >450 vs. ≤ 450 mg/L	3.78 (1.96–7.32)	<0.001	3.42 (1.72–6.82)	<0.001
Active lesion on brain stem. yes vs. no	3.28 (1.13–9.55)	0.029	3.80 (1.17–12.32)	0.026

*OR, odds ratio; CI, confidence interval; CSF, cerebrospinal fluid*.

## Discussion

Although a preventive treatment could largely reduce most NMOSD relapses, recurrent attacks are still a great medical concern (9). In this study, 197 attacks in 160 patients, treated with high-dose IVMP as a standalone therapy in the first 7 days, were identified in our NMOSD registry. In line with previous reports from smaller patient cohorts (5, 19), IVMP was ineffective in a notable proportion of NMOSD attacks (55 in 197 attacks, 27.9%). By comparing the clinical features of responders and non-responders, this study showed that patients with previous use of immunosuppressants, higher levels of CSF protein, and active lesions in the brainstem are more likely to respond poorly to high-dose IVMP alone during an acute attack. Therefore, in these patients, early treatment with steroids combined with another strategy, such as PLEX, IA, or IVIG, should be encouraged.

In the acute phase of NMOSD, prompt treatment with IVMP is the first choice (5, 7, 8, 20). For patients who harbor AQP4 antibodies, it is crucial to initiate IVMP treatment as early as possible, as even a 7-day delay in IVMP treatment can be detrimental to the recovery of vision of patients with ON (21). IVMP may inhibit the inflammatory cascade by suppressing the production of inflammatory cytokines, inhibiting T cell activation (22), and modulating different subsets of circulating B cells (23); furthermore, it promotes recovery in a large proportion of the attacks (24). In cases responding poorly to IVMP, the combination with an aggressive treatment such as PLEX, IA, or IVIG is recommended to improve the clinical outcome, eliminating pathogenic plasma factors, such as autoantibodies and complement components (20, 24, 25). A large retrospective study showed that the escalation of therapy improves the outcome in every type of NMOSD attacks, and isolated myelitis responded better to PLEX/IA than to high-dose IVMP as the initial treatment course (26). In addition, there is increasing evidence that long-term immunosuppressive therapy is essential to reduce disease activity and to avoid further attacks in the remission phase (20, 24). Our results, however, have shown that the previous use of immunosuppressants might be an independent risk factor of resistance to high-dose IVMP therapy and possibly an indicator for early initiation of aggressive therapy in acute relapses. The use of immunosuppressants, such as azathioprine or mycophenolate mofetil, which suppresses the inflammatory cascade modulating the function of different lymphocytes, largely overlaps with the therapeutic effects of IVMP.

Older age at onset was reported as a risk predictor of visual and motor disability in NMOSD (27). The late-onset course was associated with rapid disease progression and long-term disability (28–30), which might be explained by malfunctions in self-repair and blunted long-lived memory responses in aging individuals (31). Regarding responses to steroid treatment, conflicting results were reported. In a retrospective analysis of a cohort of patients with acute optic neuritis, IVMP treatment was administered, and age was found not to be associated with the outcome (32). In contrast, age at onset was reported to influence the effect of intravenous steroid pulse therapy on optic neuritis and was associated with recovery of visual acuity (33). Similarly, in the latter study, our results showed that age over 45 years old at the time of attacks could suggest an inadequate response to high-dose IVMP in NMOSD; however, this is not an independent indicator of the response in multivariable analysis. While age is not a modifiable risk factor, our results provided evidence that aggressive treatment should be considered at an early stage in patients over 45 years of age.

The serum anti-AQP4 antibody titer has been studied extensively for NMOSD monitoring, despite several different results. It has been reported that higher anti-AQP4 antibody titers in serum were associated with complete blindness and extensive lesions on MRI in patients with active NMOSD attacks (34). Other studies have shown that clinical activity and neuroinflammation are associated with the levels of AQP4-IgG titers in the CSF but not the serum (35). Some studies suggest that most AQP4-IgG are produced in peripheral tissues and that serum is the optimal and most cost-effective specimen for AQP4-IgG testing (36). However, the titer level does not reflect the ongoing disease activity or the neurological prognosis; thus, repeated follow-ups of titer levels may not be useful for the management of NMOSD patients (37). In our cohort, we tested serum AQP4-IgG titers at attack onset, and we found no difference between steroid responders and non-responders. No correlation was observed between AQP4-IgG titers and steroid response in our study.

Distinct profiles in the CSF were observed in patients with NMOSD (38–41). Biomarkers in CSF, including glial fibrillary acid protein (GFAP) and neurofilament light, were found to be associated with disease activity and disability in NMOSD (40). Elevated cytokines in CSF, such as macrophage-colony stimulating factor, interferons, interleukin-10, and soluble interleukin-2 receptor, could differentiate NMOSD from MS (39), and CNS lymphoma (41). Basic parameters in routine CSF testing were also found to be useful in clinical practice. In AQP4-IgG seropositive NMOSD patients, CSF protein concentrations were shown to be positively correlated with serum AQP4-IgG levels (38). In our study, attacks resistant to high-dose steroids tended to have higher levels of cell count and albumin quotient in the CSF than responders to IVMP. Moreover, elevated CSF protein levels were an independent risk factor for resistance, suggesting more severe blood–brain barrier disruption and increased intrathecal inflammation in non-responders than responders.

It has been shown in several studies that an initial treatment of IVMP alone may not be sufficient as the first line of therapy, especially in NMOSD with high cervical myelitis/brainstem involvement with respiratory compromise (5, 42). A retrospective analysis demonstrated that an escalation of therapy in attacks involving the spinal cord had a better outcome than high-dose steroid pulse therapy alone (2). Our study explored the features of clinical manifestations and imaging findings in attacks of IVMP non-responders. We found that active brainstem lesions in MR images could predict resistance to high-dose steroids alone, even without specific clinical manifestations. The mismatch between clinical manifestations and MR findings could be explained by the fact that clinical onset could be followed by multifocal locations of attacks. When active lesions occur in the brainstem, as first-line therapeutic approach, administering only IVMP might be insufficient.

Aggressive procedures, most notably plasma exchange, were not performed as a routine practice for patients with low EDSS at admission in our center. However, these therapies were often considered in severe NMOSD attacks, in case of insufficient response to high-dose IVMP. An improved clinical benefit of early initiation of apheresis during severe attacks of NMOSD with higher pretreatment EDSS was reported in several studies (9, 43). The factors for an effective differentiation between responders and patients resistant to IVMP remain unclear, making it difficult to determine whether early initiation of aggressive procedures should be performed, especially in non-severe attacks. On the other hand, these factors may indicate that severe attacks were more likely to require a combination treatment of second-line therapy with high-dose IVMP.

Our study has several limitations. First, it is a single-center retrospective study based on data from medical records. A prospective multicenter study is an ideal way of finding predictors in any given disease. Second, the long-term clinical efficacy of IVMP alone should be investigated. However, due to ethical issues, second-line therapy was always performed to rescue patients resistant to IVMP, making it difficult to conduct a comparative study. Although the main criterion of the patients' response to IVMP was retrospectively determined after data collection, EDSS scores were prospectively obtained during the long-term follow-up to ensure a reliable analysis in our center. Third, further research on the comparisons between rescue therapies, such as PLEX, IA, and IVIG, is needed to provide personalized precision treatment for non-responders to steroids. The correct time to initiate these aggressive procedures is another question that needs to be answered, especially for patients with non-severe attacks.

## Data Availability Statement

The data that support the findings of this study are available from the corresponding author upon reasonable request.

## Ethics Statement

The studies involving human participants were reviewed and approved by The Institutional Review Board at Tongji Hospital of Tongji Medical College, Huazhong University of Science and Technology (TJ-IRB20190502). Written informed consent to participate in this study was provided by the participants' legal guardian/next of kin.

## Author Contributions

B-TB and D-ST: had full access to all the data in the study and take responsibility for the integrity of the data and the accuracy of the data analysis. CQ, RT, S-QZ, BC, MC, D-ST, and B-TB: concept and design. CQ, RT, S-QZ, BC, MC, H-HY, Y-HC, KS, L-JW, B-TB, and D-ST: acquisition, analysis, or interpretation of data. CQ, RT, S-QZ, BC, MC, H-HY, Y-HC, and KS: drafting of the manuscript. L-JW, D-ST, and B-TB: critical revision of the manuscript for important intellectual content. RT, S-QZ, BC, MC, H-HY, Y-HC, and KS: statistical analysis. D-ST, B-TB, and L-JW: administrative, technical, or material support. D-ST and B-TB: supervision. All authors contributed to the article and approved the submitted version.

## Conflict of Interest

The authors declare that the research was conducted in the absence of any commercial or financial relationships that could be construed as a potential conflict of interest.
